# Simultaneous detection of *G6PD* mutations using SNPscan in a multiethnic minority area of Southwestern China

**DOI:** 10.3389/fgene.2022.1000290

**Published:** 2023-01-10

**Authors:** Huagui Wei, Chunfang Wang, Weiyi Huang, Liqiao He, Yaqun Liu, Huiying Huang, Wencheng Chen, Yuzhong Zheng, Guidan Xu, Liyun Lin, Wujun Wei, Weizhong Chen, Liying Chen, Junli Wang, Min Lin

**Affiliations:** ^1^ Center for Clinical Laboratory Diagnosis and Research, The Affiliated Hospital of Youjiang Medical University for Nationalities, Baise, China; ^2^ School of Biotechnology and Food Engineering, Hanshan Normal University, Chaozhou, China; ^3^ Department of Medical Laboratory, Chaozhou People’s Hospital Affiliated to Shantou University Medical College, Chaozhou, China; ^4^ School of Laboratory Medicine, Youjiang Medical University for Nationalities, Baise, China

**Keywords:** G6PD deficiency, mutation spectrum, southwestern China, SNPscan assay, *G6PD* genotype

## Abstract

**Objectives:** Baise, a multiethnic inhabited area of southwestern China, is a historical malaria-endemic area with a high prevalence of *G6PD* deficiency. However, few studies of *G6PD* deficiency have been conducted in this region. Therefore, we performed a genetic analysis of *G6PD* deficiency in the Baise population from January 2020 to June 2021.

**Methods:** A SNPscan assay was developed to simultaneously detect 33 common Chinese *G6PD* mutations. 30 *G6PD*-deficient samples were used for the method’s validation. Then, a total of 709 suspected *G6PD*-deficient samples collated from the Baise population were evaluated for *G6PD* status, type of mutation and effect of mutations.

**Results:** The SNPscan test had a sensitivity of 100% [95% confidence interval (CI): 94.87%–100%] and a specificity of 100% (95% CI: 87.66%–100%) for identifying *G6PD* mutations. A total of fifteen mutations were identified from 76.72% (544/709) of the samples. The most common mutation was discovered to be *G6PD* Kaiping (24.12%), followed by *G6PD* Canton (17.91%), and *G6PD* Gaohe (11.28%). We compared the *G6PD* mutation spectrum among Zhuang, Han and other Southeast Asian populations, and the Zhuang population’s mutation distribution was quite similar to that in the Han population.

**Conclusion:** This study provided a detailed *G6PD* mutation spectrum in Baise of southwestern China and will be valuable for the diagnosis and research of *G6PD* deficiency in this area. Furthermore, the SNPscan assay could be used to quickly diagnose these *G6PD* mutations accurately.

## 1 Introduction

Glucose-6-phosphate dehydrogenase (*G6PD*) deficiency is one of the most common enzymatic disorders of red blood cells, with a particularly high prevalence in tropical and subtropical regions, including southern China (Howell, 2006). According to the degree and extent of the enzyme deficiency, the World Health Organization (WHO) divided *G6PD* variants into four classifications in homozygous and hemizygous individuals (WHO, 2022). G6PD insufficiency manifests clinically as a range of conditions, ranging from severe enzyme deficiency to enhanced enzyme activity (Filosa et al., 1996). The most frequent clinical symptoms in patients are acute hemolysis, newborn hyperbilirubinemia, and chronic hemolysis, which are brought on by external factors including eating fava beans, taking specific medications, contracting an infection, or having a metabolic disorder (Jiang et al., 2006).

The *G6PD* gene (OMIM ID: 305900) spans 18 kb on the X chromosome (Xq28), contains an open reading frame of 1,545 bp, and encodes 515 amino acids (Tian et al., 2013; Wisnumurti et al., 2019). To date, approximately 217 mutations have been described worldwide (Gómez-Manzo et al., 2016). The *G6PD* mutation spectrum varies between different regions and ethnicities. The frequency distribution of these mutations closely correlates with populations that were exposed historically to endemic malaria (Dombrowski et al., 2017). Baise is a multiethnic inhabited area of southwestern China. The minority population accounts for 85% of the total population. It has a monsoon-influenced, humid subtropical climate and is a historical malaria-endemic area (Ji-Guang et al., 2017; Liang et al., 2020; Zheng et al., 2020). The spectrum of *G6PD* mutations, however, is poorly understood.

Currently, several analytical methods have been validated and developed to detect *G6PD* mutations, such as direct sequencing (Maloukh et al., 2021), reverse dot blot (RDB) assays (Chen et al., 2012; Duan et al., 2017; Zhang et al., 2016), high-resolution melting analysis (HRMA) (Boonyuen et al., 2021; Yang et al., 2015) and PCR-restriction fragment length polymorphism (PCR-RFLP) (Kumar et al., 2020). Although the aforementioned methods are powerful and exact, they are expensive, time-consuming and have low throughput (Zhang et al., 2016). The accuracy, sensitivity, and specificity of the SNPscan technology have been shown in numerous investigations. It is also high-throughput and cost-effective (Duan et al., 2017). Because of this, SNPscan is regarded as an acceptable method for the genetic diagnosis of G6PD deficiency.

In the present study, we established a SNPscan assay to identify 33 *G6PD* mutations. Combining the SNPscan assay with DNA sequence analysis for genotype detection and phenotypic screening, we studied the spectrum of *G6PD* mutations in Baise. Our research is essential for creating a community-based carrier screening and prevention program in the area.

## 2 Materials and methods

### 2.1 Subjects

A total of 709 suspected G6PD-deficient samples were enrolled from the Baise region of Guangxi Zhuang Autonomous Region between January 2020 and June 2021. These subjects included 346 males and 363 females, between the ages of 1 day old and ninety. Information on ethnic groups was collected. The Affiliated Hospital of Youjiang Medical University for Nationalities’ Ethics Committee accepted the study. Informed written consent was obtained from all adult participants or the guardians of pediatric participants. Ethylenediaminetetraacetic acid (EDTA) tubes were used to collect blood samples, which were then brought to the lab and kept in storage at 4°C.

### 2.2 Quantitative G6PD enzyme activity

The G6PD enzyme activity was measured by a commercial G6PD Detection kit (Korfang Biotechnology Co., Guangzhou, Guangdong, China) according to the rate method (Zhong et al., 2018), which was approved by the China Food and Drug Administration (CFDA) (reg. no. CFDA (P) 20193400771). According to the National Inspection Operational Regulations, 1 mL solution (Korfang Biotechnology Co., Guangzhou, Guangdong, China) was added to a small cup, and then 20 μL of erythrocyte was accurately absorbed into the solution without the plasma layer. The activity of G6PD was detected by the rate method on Hitachi 7170A automatic biochemical analyzer (HITACHI, Japan), and the concentration of hemoglobin in hemolysis was detected by the HiCN method. This method can detect NADPH production in fixed time, which reflect G6PD activity in red blood cells. In each test run, the accuracy of the test findings was checked by calibration and the use of controls offered by KOFA Medical. The reference range of adults with values below 1.30 KU/L (1.30–3.60) and infants with values below 1.70 KU/L (1.70–4.00).

### 2.3 Genomic DNA extraction

According to the manufacturer’s recommendations, genomic DNA was extracted from all samples using a QIAamp DNA Blood Mini kit (Qiagen, Hilden, Germany). The DNA concentration was measured using a Thermo Scientific Nanodrop™ 2000 spectrophotometer and subsequently adjusted to 50 ng/L.

### 2.4 SNPscan assay for *G6PD* mutations

**FIGURE 1:**
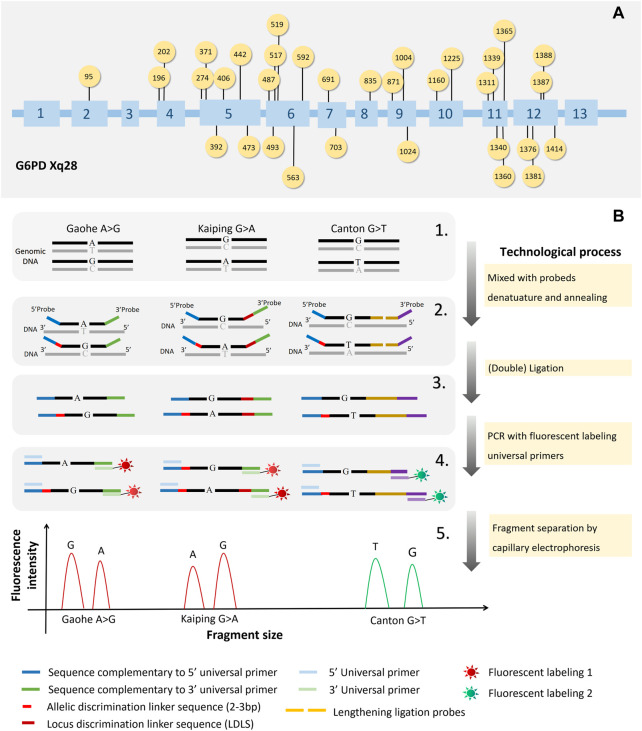
The workings of SNPscan technology and the locations of 33 *G6PD* gene mutations. The locations of the 33 mutations in the *G6PD* gene are shown in **(A)**. The principles of SNPscan technology are shown in **(B)**.

A multiplex SNPscan assays were designed to detect 33 *G6PD* mutations reported in Chinese population (Wang et al., 2021) as follow: *G6PD* Gaohe (c.95A>G), *G6PD* Songklanagarind (c.196T>A), *G6PD* Asahi (c.202G>A), *G6PD* Chinese-4 (c.392G>T), *G6PD* Valladolid (c.406C>T), *G6PD* Liuzhou (c.442G>A), *G6PD* Shenzhe (c.473G>A), *G6PD* Mahidol (c.487G>A), *G6PD* Taipei (c.493A>G), *G6PD* Nankang (c.517T>C), *G6PD* Miaoli (c.519C>T/G), *G6PD* Mediterranean (c.563C>T), *G6PD* Shunde (c.592C>T), *G6PD* Nanning (c.703C>T), *G6PD* Haikou (c.835A>G/T), *G6PD* Viangchan (c.871G>A), *G6PD* Fushan (c.1004C>A/T), *G6PD* Chinese-5 (c.1024C>T), *G6PD* Beverly Hills (c.1160G>A), *G6PD* Santiago de Cuba (c.1339G>A), *G6PD* Jiangxi (c.1340G>T), *G6PD* Union (c.1360C>T), *G6PD* Canton (c.1376G>T), *G6PD* Yannan (c.1381G>A), *G6PD* Kamiube (c.1387C>T), *G6PD* Kaiping (c.1388G>A), *G6PD* Laibin (c.1414A>C), and four unnamed mutations (c.274C>T, c.371A>G, c.691G>C and c.1225C>T) and two Silent mutation (c.1311C>T, c.1365-13T>C). The 33 *G6PD* mutation sites in the *G6PD* gene are shown in Figure 1A.

As shown in Figure 1B, previously mentioned, the double ligation and multiplex fluorescence PCR serves as the foundation for the SNPscan test. (Wei et al., 2013). The primers and probes are listed in Supplementary Table S1. For each SNPscan assay, 12 µL of ligation mixture was first prepared to contain 2 μL of 10 × ligase buffer, 1 μL of 1 × probe mix, .5 μL of ligase, 7 μL of ddH_2_O and 1 μL of 30–250 ng of DNA sample. The ligation reaction was performed on an ABI 2720 thermal cycler with the following cycling program: 98°C for 2 min; 5 cycles of 95°C for 1 min, 58°C for 3 h; 94°C for 2 min, hold at 72°C. Fluorescence in multiplex After that, PCRs were run on each ligation product. Every PCR mixture was made in 20 μL containing 2× PCR Buffer, 1 μL of primer mix, 8 μL of ddH_2_O, and 1 μL of ligation product. The PCR program was as follows: 95°C for 2 min; 9 cycles of 94°C for 20 s, 62°C–.5°C/cycle for 40 s, and 72°C for 1.5 min; 26 cycles of 94°C for 20 s, 58°C for 40 s, and 72°C for 1.5 min; 60°C for 1 h; and hold at 4°C. Using a capillary electrophoresis system and an ABI 3730XL sequencer, PCR products were separated and identified. Raw data were analysed with GeneMapper 4.1 software (Applied Biosystems, United States), and the genotypes of each locus were determined.

### 2.5 DNA sequencing

In order to confirm the SNPscan assay results, PCR amplification and DNA sequencing of the entire *G6PD* coding region was performed as described in our earlier research (Pan et al., 2013; Zheng et al., 2020). Purification and sequencing of PCR products were done by Shanghai Vebery Biotechnology (Shanghai, China). All primers are in Supplementary Table S4 (Pan et al., 2013).

### 2.6 Bioinformatics analysis of *G6PD* mutations

The bioinformatics software used in this work was used to analyze each *G6PD* mutation identified. Moreover, the pI of *G6PD* variants (i.e., monomers) was determined using Kozlowski’s protein isoelectric point (IP) calculator (http://isoelectric.org/). Utilizing ConSurf (http://bental.tau.ac.il/new_ConSurfDB/), we looked at the evolutionary conservation of mutant amino acid residues. The pathogenicity of these potential variants was assessed by PolyPhen-2 (Polymorphism Phenotyping v2) (http://genetics.bwh.harvard.edu/pph2/) and Sorting Intolerant from Tolerant (SIFT) web server (http://sift.jcvi.org) prediction models.

### 2.7 Statistical analysis

The data are collated in Excel. All data were statistical using SPSS 22.0. Descriptive statistics were used to estimate the accuracy.

## 3 Results

### 3.1 Development and validation of the SNPscan assay

**FIGURE 2:**
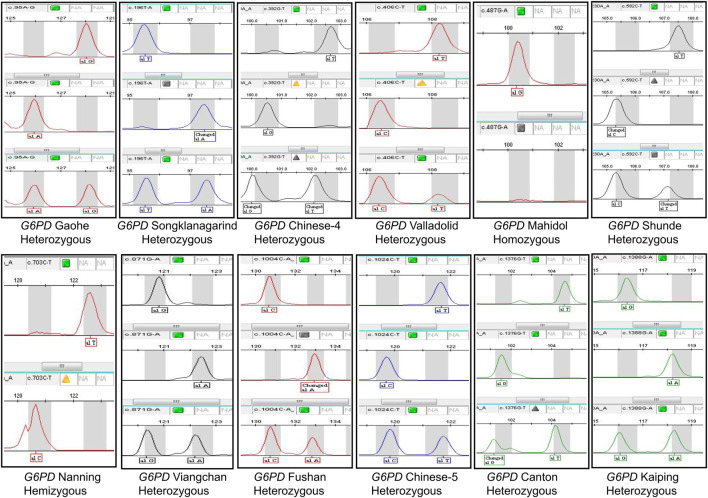
The results of *G6PD* positive mutation detected by SNPscan technology.

A SNPscan assay was developed to detect 33 *G6PD* mutations reported in Chinese individuals. As shown in Figure 1, it could precisely distinguish heterozygous mutations and homozygous/hemizygous mutations by capillary electrophoresis (Figure 2). To confirm the accuracy of the SNPscan assay, 30 samples were blindly analysed using PCR amplification and DNA sequencing (Supplementary Table S2). Comparatively speaking to direct DNA sequencing, the SNPscan assay was 100% sensitive [95% confidence interval (CI): 94.87–100%] and 100% specific (95% CI: 87.66–100%), without any cross-reactivity for the identification of *G6PD* mutations. Additionally, the SNPscan assay could precisely distinguish double mutations, such as Canton/Viangchan, Gaohe/Kaiping and Canton/Kaiping. The created approach is dependable for identifying *G6PD* mutations, according to all of the evidence, detailed data are shown in Supplementary Table S3.

### 3.2 Mutation spectrum of G6PD deficiency

Fifteen *G6PD* mutations were identified by the SNPcan assay in the Baise population (Table 1). Among the 709 G6PD-deficient people, 544 (277 females and 267 males) had at least one mutation in the *G6PD* gene. Among the 277 females with mutated G6PD deficiency, we identified 22 (3.10%) homozygotes and 255 heterozygotes, including 73 compound heterozygotes. The mutations of *G6PD* Kaiping, *G6PD* Canton and *G6PD* Gaohe were the three dominant mutations with an overall frequency of higher than 79.06%, followed by *G6PD* Chinese-5, *G6PD* Viangchan and *G6PD* Valladolid, with a frequency of 2.11% as a minimum, respectively. The number and frequency of various mutations are presented in Table 1.

**TABLE 1:** Frequency of all *G6PD*-positive mutations and predicted consequences before and after amino acid changes.

Name	Mutation	Protein	PolyPhen-2	PROVEAN	SIFT	FoldX (stability)	PI	Total (*n*)	Frequency (%)
Gaohe	c.95 A>G	p.His32Arg	PROBABLY DAMAGING	Deleterious	Tolerated	-.583907	6.19	90	17.25
Songklanagarind	c.196 T>A	p.Phe66Ile	BENIGN	Neutral	Tolerated	.58045	6.10	2	.38
NR	c.274 C>T	p.Pro92Ser	BENIGN	Neutral	Tolerated	1.66913	6.10	1	.19
Chinese-4	c.392 G>T	p.Gly131Val	PROBABLY DAMAGING	Deleterious	Damaging	29.6132	6.10	3	.58
Valladolid	c.406 C>T	p.Arg136Cys	PROBABLY DAMAGING	Deleterious	Damaging	2.53579	5.98	11	2.11
Mahidol	c.487 G>A	p.Gly163Ser	POSSIBLY DAMAGING	Deleterious	Damaging	7.96808	6.10	2	.38
Miaoli	c.519 C>T	p.Phe173Leu	PROBABLY DAMAGING	Deleterious	Damaging	1.35032	6.10	7	1.34
Shunde	c.592 C>T	p.Arg198Cys	PROBABLY DAMAGING	Deleterious	Damaging	4.70525	5.99	3	.58
Nanning	c.703 C>T	p.Leu235Pro	PROBABLY DAMAGING	Deleterious	Damaging	6.46278	6.10	2	.38
Viangchan	c.871 G>A	p.Val291Met	PROBABLY DAMAGING	Deleterious	Damaging	-1.19782	6.10	24	4.61
Fushan	c.1004 C>A	p.Ala335Asp	POSSIBLY DAMAGING	Neutral	Damaging	1.44222	6.10	8	1.54
Chinese-5	c.1024 C>T	p.Leu342Phe	BENIGN	Neutral	Tolerated	3.94837	6.10	45	8.64
Union	c.1360 C>T	p.Arg454Cys	PROBABLY DAMAGING	Deleterious	Damaging	2.33769	5.98	1	.19
Canton	c.1376 G>T	p.Arg459Leu	PROBABLY DAMAGING	Deleterious	Tolerated	-.424977	5.99	137	26.30
Kaiping	c.1388 G>T	p.Arg463His	PROBABLY DAMAGING	Deleterious	Damaging	.798808	6.02	185	35.51

NR: class not reported.

**FIGURE 3:**
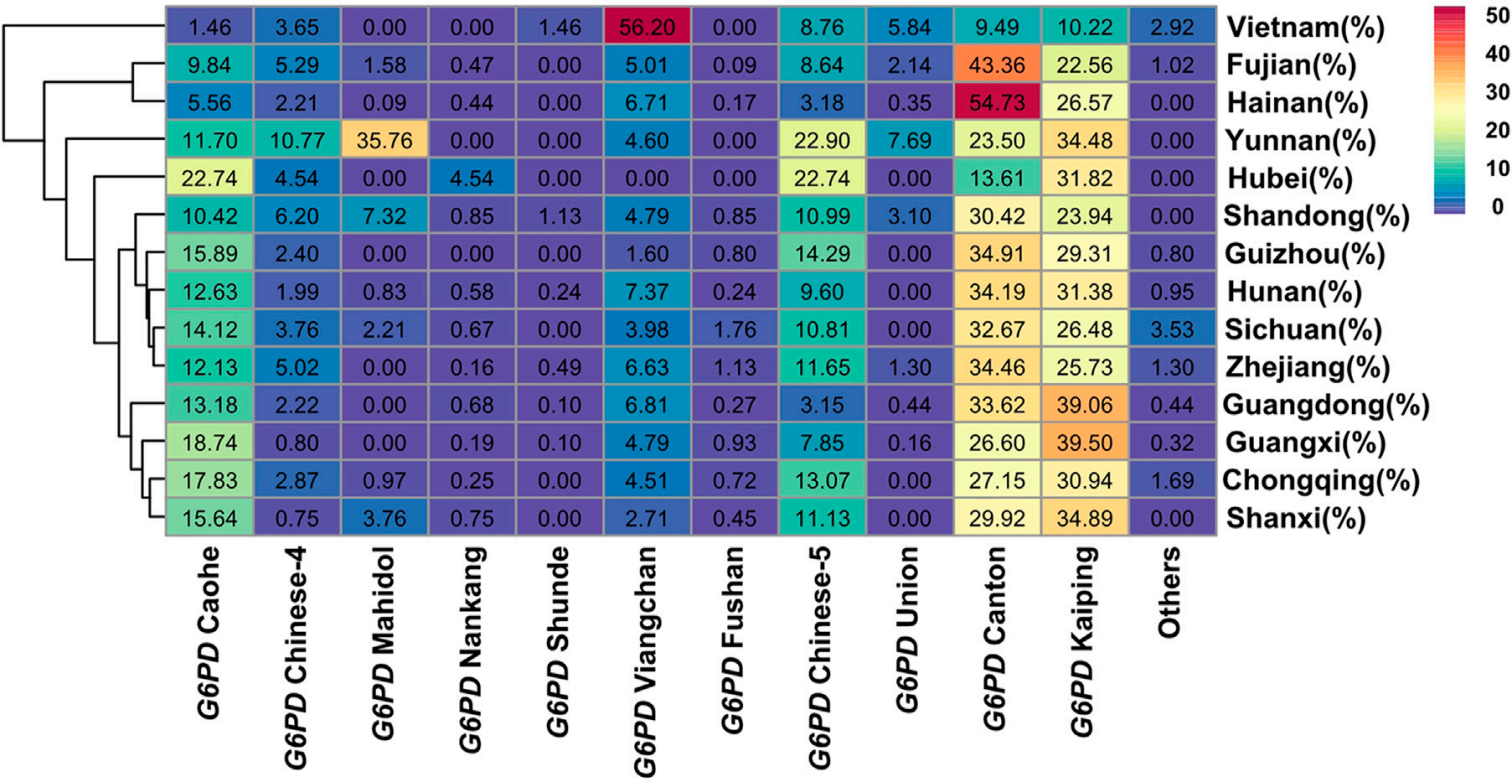
Heatmap of G6PD-deficient allele frequency distributions for Baise populations and others cities populations. Red represents the highest G6PD-deficient allele frequency, while purple represents the lowest.

These 709 samples were classified by ethnicity. There were 412 allele mutations and 15 variants in the Zhuang nationality, most of which were *G6PD* Kaiping, *G6PD* Canton and *G6PD* Gaohe, accounting for 79.08% (Table 2). However, there were only 87 (16.70%) allele mutations in the Han nationality. In addition, in order to examine the association of the major G6PD-deficient alleles in Chinese people and Southern Asian populations, data from our research or other studies were further analysed (Liu et al., 2020; Zheng et al., 2020). As shown in Figure 3, the frequencies of different G6PD-deficient alleles in different regions were plotted on a heatmap. The color of each block varies with the corresponding frequency. Purple represented the lowest allele frequency on the color scale, which went up to red for the greatest allele frequency. Obviously, Four G6PD-deficient alleles (Canton, Kaiping, Gaohe and Chinese-5) were present in relatively high frequencies in Chinese people, whereas *G6PD* Viangchan and *G6PD* Kaiping were prevalent in Southern Asian populations (Vietnam populations).

**TABLE 2:** The 709 samples were classified by ethnicity.

Name	Mutation	Zhuang (n, %)	Han (n, %)	Yao (n, %)	Buyi (n, %)	Mulao (n, %)	Total (n, %)
Caohe	c.95 A>G	76 (18.45)	9 (10.34)	0	4 (25.00)	1 (100)	90 (17.25)
Songklanagarind	c.196 T>A	1 (.24)	1 (1.15)	0	0	0	2 (.38)
NR	c.274 C>T	0	1 (1.15)	0	0	0	1 (.19)
Chinese-4	c.392 G>T	2 (.49)	1 (1.15)	0	0	0	3 (.58)
Valladolid	c.406 C>T	9 (2.18)	2 (2.30)	0	0	0	11 (2.11)
Mahidol	c.487 G>A	1 (.24)	1 (1.15)	0	0	0	2 (.38)
Miaoli	c.519 T>G	3 (.73)	2 (2.30)	0	2 (12.5)	0	7 (1.34)
Shunde	c.592 C>T	2 (.49)	1 (1.15)	0	0	0	3 (.58)
Nanning	c.703 C>T	1 (.24)	1 (1.15)	0	0	0	2 (.38)
Viangchan	c.871 G>A	19 (4.61)	5 (5.75)	0	0	0	24 (4.61)
Fushan	c.1004 C>A	7 (1.70)	0	0	1 (6.25)	0	8 (1.54)
Chinese-5	c.1024 C>T	32 (7.77)	10 (11.49)	2 (40)	1 (6.25)	0	45 (8.64)
Union	c.1360 C>T	0	1 (1.15)	0	0	0	1 (.19)
Canton	c.1376 G>T	113 (27.43)	17 (19.54)	3 (60)	4 (25.00)	0	137 (26.30)
Kaiping	c.1388 G>T	146 (35.44)	35 (40.23)	0	4 (25.00)	0	185 (35.51)
	Total	412 (100)	87 (100)	5 (100)	16 (100)	1 (100)	521 (100)

### 3.3 Effect of mutations on disease manifestation

**FIGURE 4:**
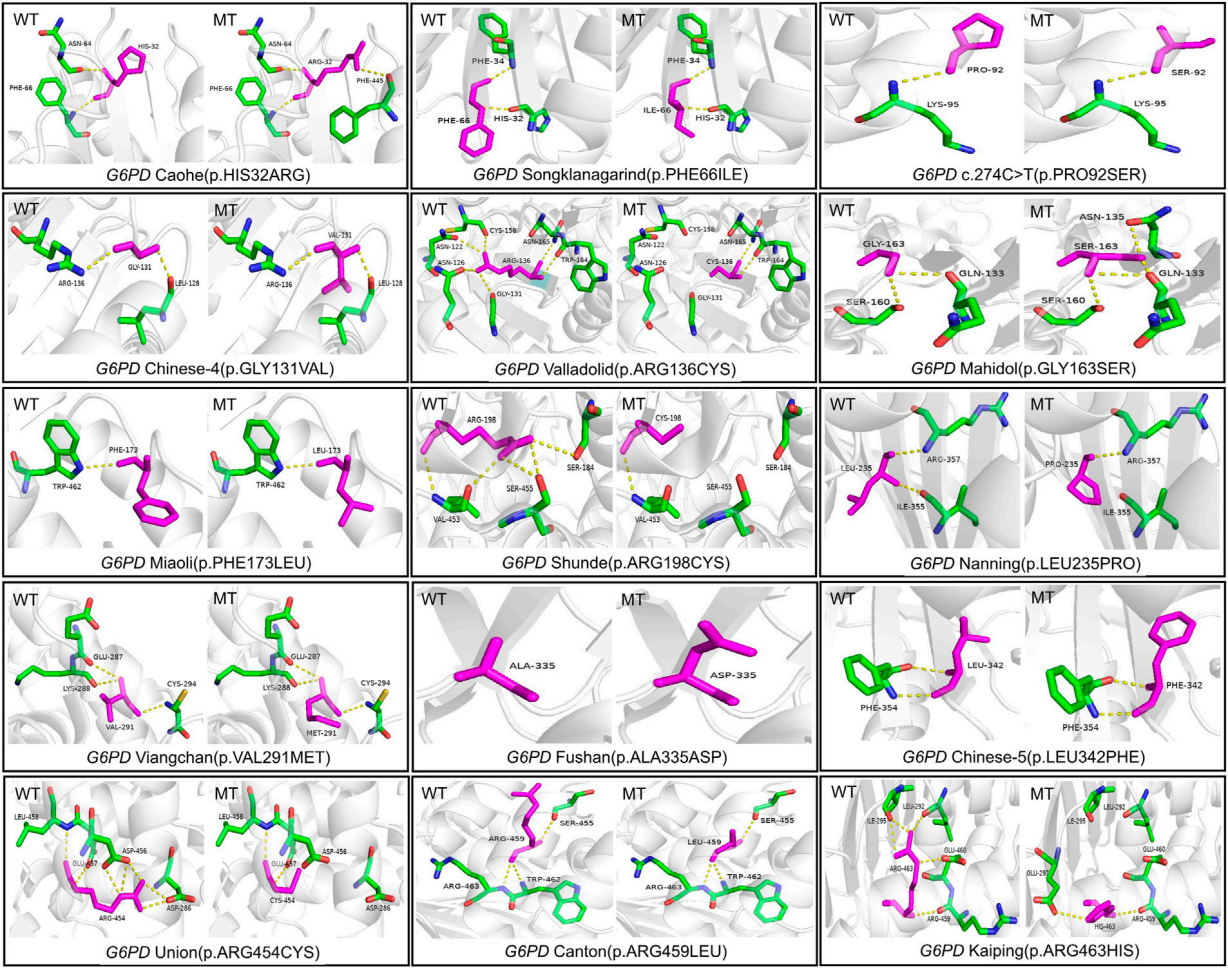
Changes in protein structure and polar bonds before and after *G6PD* mutation.

Tools from bioinformatics were used to forecast how changing an amino acid might affect how a protein function (Table 1). According to PolyPhen2.0, all variants were identified as potentially damaging (prediction score close to 1) except for *G6PD* Songklanagarind, *G6PD* c.274C>T and *G6PD* Chinese-5), similar to the results predicted by PROVEAN (except *G6PD* Fushan). However, SIFT predicted that five missense mutations (*G6PD* Gaohe, *G6PD* Songklanagarind, *G6PD* c.274C>T, *G6PD* Chinese-5, and *G6PD* Canton) could be tolerated, and the rest were damaging. FoldX was used to predict changes in the protein stability of *G6PD* (Table 1), and three variants (*G6PD* Gaohe, *G6PD* Viangchan and *G6PD* Canton) were found to increase the stability of the G6PD protein, while other missense variants were predicted to destabilize the G6PD protein. Additionally, Table 1 provides an overview of the expected pI values for each of the 15 *G6PD* variations. The changes in protein structure and polar bonds before and after *G6PD* mutation are shown in Figure 4.

## 4 Discussion

In this study, we looked studied the distribution of different *G6PD* gene variants, the prevalence of G6PD deficiency, and the relationship between genotypes and phenotypes related to enzyme function in Baise, Guangxi Zhuang Autonomous Region. The results showed that six of the most prevalent mutations were *G6PD* Kaiping, *G6PD* Canton, *G6PD* Gaohe, *G6PD* Chinese-4, *G6PD* Viangchan and *G6PD* Chinese-5, accounting for more than 60% of G6PD-deficient alleles. This result is consistent with LinZou’s research (Liu et al., 2020). The sexes and different sorts of mutation patterns affected how G6PD activities were distributed (Driscoll and Migeon, 1990). These findings present a more precise and thorough characterization of G6PD deficiency in Baise, Guangxi.

The prevalence of G6PD deficiency varies widely by region in China, with northern China having a relatively lower prevalence than southern China. G6PD deficiency was present in 2.1% of China’s population overall (He et al., 2020), and over 35 different *G6PD* gene mutations were known, with *G6PD* Kaiping and *G6PD* Canton predominating in earlier investigations (Liu et al., 2020; Jiang et al., 2006). Africa, Asia, southern Europe, the Middle East, Southeast Asia, and Mediterranean nations have the highest prevalence rates, according to reports (He et al., 2020; Liu et al., 2020). In India, in various population groups, it was discovered that 8.5% of people have G6PD deficiencies (Saravu et al., 2016). The prevalence rate varies between the tribal groupings, ranging from 2.3% to 27.0%, with an overall incidence of 7.7% (Mukherjee et al., 2015). In contrast to southern India, where it is continuously low except in the states of Andhra Pradesh and Tamil Nadu, the frequency of the G6PD-deficient allele is higher in northern and western India (Devendra et al., 2020). In Indian caste groupings, *G6PD* Mediterranean was discovered to be the most prevalent variation (Devendra et al., 2020; Sukumar et al., 2004). However, *G6PD* Kaiping was found to be the most common variant in China (Lin et al., 2018; Liu et al., 2020; Yan et al., 2010). In Southeast Asia, G6PD deficiency is diverse, as previously demonstrated by epidemiological and molecular research (Louicharoen and Nuchprayoon, 2005). In Thais, Laotians, Cambodians, and Malaysian Malays, *G6PD* Viangchan appears to be the most prevalent form (Ainoon et al., 2003; Iwai et al., 2001; Louicharoen and Nuchprayoon, 2005; Nuchprayoon et al., 2002), while the most prevalent form of G6PD in the population of Myanmar is Mahidol (Matsuoka et al., 2004). In the current research, a total of 15 harmful mutations were found, which were dominated by *G6PD* Kaiping and *G6PD* Canton, accounting for approximately 42% of all G6PD-deficient alleles. However, it is lower than previous research results (84.1%, 75.3%) in the Guangxi population (Fu et al., 2018; Yan et al., 2006). This can be because we only collected a small number of samples or because geographical disparities exist. In addition, we are a region with a high prevalence of thalassemia, moreover, hemolysis and anemia are common (Lin et al., 2015). Medication, hemolysis, and anemia can affect the detection of G6PD activity (Nuinoon et al., 2022; Pfeffer et al., 2022). In our study, these may be one of the reasons that the 165 samples with no detection of any of the 33 common mutations. However, they were detected with G6PD activity deficiency. Certainly, the other reason is that they may have rare mutations (besides 33 common mutations).

G6PD was first described by Carson in 1956 (ALVING et al., 1956). Its clinical manifestations include fulminant hemolysis, severe hyperbilirubinemia, and kernicterus, which contribute to neonatal neurological injury and risk of death (He et al., 2020; Kaplan et al., 2015; Liu et al., 2020). This condition may be brought on by infections, specific foods (such as fava beans), oxidizing medicines, and/or specific herbal therapies (Liu et al., 2020). To date, after a newborn’s screening results in a positive result, the most effective treatment for this illness is to prevent hemolysis by avoiding some oxidative stressors (Liu et al., 2020). Therefore, the general survey of G6PD deficiency, early detection and early prevention are important measures to prevent and treat the disease. There are three common measures to prevent the disease, the most important is to avoid accidental ingestion of fava beans (Reading et al., 2016); secondly, avoid taking anti-malarial drugs (primaquine, chloroquine, malaria quinine, pentaquine and adipine), sulfones (thiazole sulfone, aminophene sulfone), sulfonamides (sulfamethoxazole, sulfadimethoxine, sulfapyridine and salazosulfapyridine) and antipyretics (acetazolamide and acetanilide) and so on (http://www.g6pd.org) (Chu and Freedom, 2019; Reading et al., 2016). Finally, when the patient has an infection (viral hepatitis, influenza, pneumonia, typhoid), which should immediately seek medical help to avoid hemolysis.

Today’s G6PD deficiency diagnosis primarily uses the enzyme activity detection assay, and the main diagnosis used to avoid oxidative hemolysis cannot be other than a phenotypic test, especially in women; however, there is an added value in *G6PD* genotyping, different sorts of mutations can result in various classes of variations and exhibit various symptoms (Beutler et al., 2002; WHO, 2022). So, to establish a certain diagnosis of G6PD insufficiency, genotyping of *G6PD* mutations is beneficial (Jiang et al., 2006). In addition, the analysis of *G6PD* genotypes contributes to the study of molecular biology and genetic characterization of human populations (Hamali, 2021; Lee et al., 2022). Aside from this, the genotyping of G6PD deficiency also has a significant impact on the field’s understanding of the disorder (Li et al., 2008). The SNPscan assay used in the study covered 33 common mutations in the Chinese population and could identify more than 95% of G6PD deficiencies. Based on the detection of SNP loci, SNPscan technology can simultaneously type multiple SNP loci in one detection process (Yu et al., 2021). Numerous investigations have shown that it has good accuracy, sensitivity, and specificity and is cost-effective and high-throughput (Du et al., 2014; Yin et al., 2014; Zhang et al., 2016). Compared with the direct sequencing method, it saves more tedious operations in the experimental process, can detect multiple sites in multiple samples at the same time, and reduces the cost (Zhang et al., 2016). Compared with the gene chip method, SNPscan technology has more detection sites, so it can be flexibly designed for known target gene mutation sites and achieves high throughput (Chen et al., 2012; Duan et al., 2017; Hu et al., 2015; Zhang et al., 2016). In addition, we investigated a general comparison of costs associated with these different techniques and found that the SNPscan technique has the lowest cost (SNPscan technology: $14.26/sample, direct sequencing method: $20.97/sample, gene chip method: $69.93/sample). Therefore, a trustworthy, quick, and affordable method for identifying *G6PD* point mutations would be beneficial to patients, their families, the doctors who treat them, and the testing labs.

## Data Availability

The original contributions presented in the study are included in the article/Supplementary Material, further inquiries can be directed to the corresponding authors.
