# Supplementary material for: Simultaneous detection of G6PD mutations using SNPscan in a multiethnic minority area of Southwestern China
Source: Front Genet. 2023 Jan 10;13:1000290. doi: 10.3389/fgene.2022.1000290 (PMC9871378; doi:10.3389/fgene.2022.1000290)
Supplement: Supplementary file 1 [file DataSheet1.zip › Suppl. Table 1 (2).DOCX]

Table 1 Frequency of all *G6PD*-positive mutations and predicted consequences before and after amino acid changes.

| Name | Mutation | Protein | PolyPhen-2 | PROVEAN | SIFT | FoldX (Stability) | PI | Total (*n*) | Frequency (%) |
| --- | --- | --- | --- | --- | --- | --- | --- | --- | --- |
| Gaohe | c.95 A>G | p.His32Arg | PROBABLY DAMAGING | Deleterious | Tolerated | -0.583907 | 6.19 | 90 | 17.25 |
| Songklanagarind | c.196 T>A | p.Phe66Ile | BENIGN | Neutral | Tolerated | 0.58045 | 6.10 | 2 | 0.38 |
| NR | c.274 C>T | p.Pro92Ser | BENIGN | Neutral | Tolerated | 1.66913 | 6.10 | 1 | 0.19 |
| Chinese-4 | c.392 G>T | p.Gly131Val | PROBABLY DAMAGING | Deleterious | Damaging | 29.6132 | 6.10 | 3 | 0.58 |
| Valladolid | c.406 C>T | p.Arg136Cys | PROBABLY DAMAGING | Deleterious | Damaging | 2.53579 | 5.98 | 11 | 2.11 |
| Mahidol | c.487 G>A | p.Gly163Ser | POSSIBLY DAMAGING | Deleterious | Damaging | 7.96808 | 6.10 | 2 | 0.38 |
| Miaoli | c.519 C>T | p.Phe173Leu | PROBABLY DAMAGING | Deleterious | Damaging | 1.35032 | 6.10 | 7 | 1.34 |
| Shunde | c.592 C>T | p.Arg198Cys | PROBABLY DAMAGING | Deleterious | Damaging | 4.70525 | 5.99 | 3 | 0.58 |
| Nanning | c.703 C>T | p.Leu235Pro | PROBABLY DAMAGING | Deleterious | Damaging | 6.46278 | 6.10 | 2 | 0.38 |
| Viangchan | c.871 G>A | p.Val291Met | PROBABLY DAMAGING | Deleterious | Damaging | -1.19782 | 6.10 | 24 | 4.61 |
| Fushan | c.1004 C>A | p.Ala335Asp | POSSIBLY DAMAGING | Neutral | Damaging | 1.44222 | 6.10 | 8 | 1.54 |
| Chinese-5 | c.1024 C>T | p.Leu342Phe | BENIGN | Neutral | Tolerated | 3.94837 | 6.10 | 45 | 8.64 |
| Union | c.1360 C>T | p.Arg454Cys | PROBABLY DAMAGING | Deleterious | Damaging | 2.33769 | 5.98 | 1 | 0.19 |
| Canton | c.1376 G>T | p.Arg459Leu | PROBABLY DAMAGING | Deleterious | Tolerated | -0.424977 | 5.99 | 137 | 26.30 |
| Kaiping | c.1388 G>T | p.Arg463His | PROBABLY DAMAGING | Deleterious | Damaging | 0.798808 | 6.02 | 185 | 35.51 |

NR: Class not reported.
