# Supplementary material for: Simultaneous detection of G6PD mutations using SNPscan in a multiethnic minority area of Southwestern China
Source: Front Genet. 2023 Jan 10;13:1000290. doi: 10.3389/fgene.2022.1000290 (PMC9871378; doi:10.3389/fgene.2022.1000290)
Supplement: Supplementary file 1 [file DataSheet1.zip › Suppl. Table 2.DOCX]

Table 2 The 709 samples were classified by ethnicity.

| Name | Mutation | Zhuang (n, %) | Han (n, %) | Yao (n, %) | Buyi (n, %) | Mulao (n, %) | Total (n, %) |
| --- | --- | --- | --- | --- | --- | --- | --- |
| Caohe | c.95 A>G | 76 (18.45) | 9 (10.34) | 0 | 4 (25.00) | 1 (100) | 90 (17.25) |
| Songklanagarind | c.196 T>A | 1 (0.24) | 1 (1.15) | 0 | 0 | 0 | 2 (0.38) |
| NR | c.274 C>T | 0 | 1 (1.15) | 0 | 0 | 0 | 1 (0.19) |
| Chinese-4 | c.392 G>T | 2 (0.49) | 1 (1.15) | 0 | 0 | 0 | 3 (0.58) |
| Valladolid | c.406 C>T | 9 (2.18) | 2 (2.30) | 0 | 0 | 0 | 11 (2.11) |
| Mahidol | c.487 G>A | 1 (0.24) | 1 (1.15) | 0 | 0 | 0 | 2 (0.38) |
| Miaoli | c.519 T>G | 3 (0.73) | 2 (2.30) | 0 | 2 (12.5) | 0 | 7 (1.34) |
| Shunde | c.592 C>T | 2 (0.49) | 1 (1.15) | 0 | 0 | 0 | 3 (0.58) |
| Nanning | c.703 C>T | 1 (0.24) | 1 (1.15) | 0 | 0 | 0 | 2 (0.38) |
| Viangchan | c.871 G>A | 19 (4.61) | 5 (5.75) | 0 | 0 | 0 | 24 (4.61) |
| Fushan | c.1004 C>A | 7 (1.70) | 0 | 0 | 1 (6.25) | 0 | 8 (1.54) |
| Chinese-5 | c.1024 C>T | 32 (7.77) | 10 (11.49) | 2 (40) | 1 (6.25) | 0 | 45 (8.64) |
| Union | c.1360 C>T | 0 | 1 (1.15) | 0 | 0 | 0 | 1 (0.19) |
| Canton | c.1376 G>T | 113 (27.43) | 17 (19.54) | 3 (60) | 4 (25.00) | 0 | 137 (26.30) |
| Kaiping | c.1388 G>T | 146 (35.44) | 35 (40.23) | 0 | 4 (25.00) | 0 | 185 (35.51) |
|  | Total | 412 (100) | 87 (100) | 5 (100) | 16 (100) | 1 (100) | 521 (100) |
